# Endoscopic indigo carmine spraying for evaluation of intestinal mucosal permeability: Prospective pilot study

**DOI:** 10.1055/a-2697-7599

**Published:** 2025-09-29

**Authors:** Hirokazu Fukui, Shojiro Kikuchi, Noriyuki Ojima, Tomonori Yokoyama, Masataka Ikeda, Shinichiro Shinzaki

**Affiliations:** 1 12818 Department of Gastroenterology, Hyogo Medical University, Nishinomiya, Japan; 2 12818 Department of Omics Medicine, Hyogo Medical University, Nishinomiya, Japan; 3 12818 Laboratory of Medical Frontiers, Institution for advanced Medical Sciences, Hyogo Medical University, Nishinomiya, Japan; 4 65828 Analytical and Measuring Instruments Division, Shimadzu Corporation, Kyoto, Japan; 5 12818 Division of Lower Gastrointestinal Surgery, Department of Gastroenterological Surgery, Hyogo Medical University, Nishinomiya, Japan

**Keywords:** Endoscopy Lower GI Tract, Inflammatory bowel disease, Diagnosis and imaging (inc chromoendoscopy, NBI, iSCAN, FICE, CLE...)

## Abstract

**Background and study aims:**

"Leaky gut," caused by increased mucosal permeability, plays a pivotal role in various diseases. However, few methods are available to evaluate intestinal mucosal permeability in the living human body. We established a novel method for evaluation of mucosal permeability using indigo carmine (IC).

**Patients and methods:**

Subjects undergoing colonic endoscopy for screening of colon polyps or evaluation of ulcerative colitis (UC) severity were enrolled. IC was endoscopically sprayed in the cecum, and blood samples were obtained before spraying and at 30 and 60 minutes after. Serum IC level was analyzed by liquid chromatographer/mass spectrometer equipped with a Nexera HPLC system.

**Results:**

In both the control (subjects screened for colon polyps) and UC groups, all subjects had their highest serum IC levels at 30 minutes after spraying. Serum IC level was significantly higher in UC patients than in the controls at both 30 and 60 minutes after spraying. In the UC group, serum IC levels at both 30 and 60 minutes were significantly higher in patients with a Mayo endoscopic subscore (MES) 1 at the cecum than in those with MES 0 in the same area.

**Conclusions:**

Endoscopic spraying with IC is useful for evaluation of intestinal mucosal permeability.

## Graphical abstract

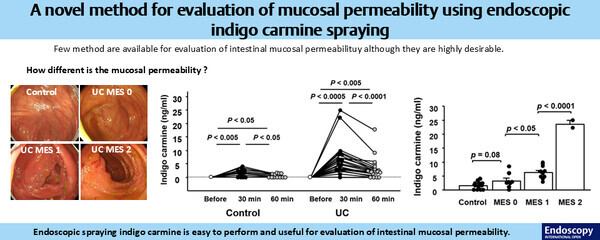

## Introduction

Microbiota of the gut and its related intestinal environment are known to play a pivotal role in pathophysiology of various diseases 1 2 3 . Numerous researchers have speculated that increased mucosal permeability due to impairment of intestinal barrier function, so called “leaky gut,” is a first step in development of an abnormal intestinal environment, leading to harmful disease-related pathophysiology 4 5 6 . Cumulative evidence suggests that intestinal mucosal permeability is increased in animal models of various gut conditions such as inflammatory bowel disease 7 8 or drug/food/psychological stress-induced dysbiosis 9 10 11 . However, it is very difficult to prove whether increased mucosal permeability actually plays a critical role in pathophysiology of human diseases because few methods are available for evaluation of intestinal mucosal permeability in the living human body. In this context, it is highly desirable to establish a reliable method for evaluation of human intestinal mucosal permeability for investigation of disease mechanisms and pathophysiology. In the present study, we attempted to establish a method for evaluation of mucosal permeability with indigo carmine (IC), a dye used for gastrointestinal endoscopy worldwide.

## Patients and methods

### Subjects

Patients undergoing colonic endoscopy for screening colon polyps (n = 12) or assessment of ulcerative colitis (UC) severity (n = 20) were enrolled. We hypothesized that patients with UC would be suitable as positive controls in view of their increased intestinal permeability, whereas patients undergoing colon polyp screening would be suitable as negative controls because their intestinal permeability is similar to that of healthy individuals, or at least not as increased as that in UC. Only patients with clinically and endoscopically confirmed UC 12 were included. In both groups, individuals with a history of gastrointestinal surgery, systemic inflammatory disease, chemotherapy, and management of malignancies were excluded.

### Endoscopic procedure and blood sampling

After observation of the cecum and ascending colon, IC (20 mg/20 mL distilled water) was sprayed in the cecum, then left without aspiration. Thereafter, routine endoscopy was performed. Blood samples were obtained intravenously (IV) before spraying IC, and at 30 and 60 minutes after.

Data about age, sex, and blood biochemistry were collected for each subject. For UC patients, data also included disease duration, disease activity, disease type and medications. UC disease activity was evaluated using the Mayo score 13 as follows: remission, 0–2; mild, 3–5; moderate, 6–10; severe, 11–12. Degree of colonic mucosal injury at the cecum, but not at the portion with severest injury, was graded according to the Mayo endoscopic subscore (MES) 14 .

### Ethical considerations

This study was performed with approval (No. 202405–910) from the Ethics Committee of Hyogo Medical University. All patients provided written informed consent before participation and the study was conducted according to the principles governing human research stipulated by the Declaration of Helsinki.

### Analysis of serum indigo carmine level

Serum was separated from each blood sample, then to 20 µL of serum, 100 µL of methanol containing 100 ng/mL Orange G was added, and the mixture was centrifuged at 12,000 x g for 10 minutes at 4°C. Then, 80 µL of supernatant was passed through MonoSpin Phospholipid (GL Science, Tokyo, Japan), the eluate was vacuum-dried, and redissolved in 150 µL of ultrapure water for LC/ MS analysis. Quantitative analysis of serum IC (range, 1–100 ng/mL) was performed using an LCMS-8060 triple quadrupole mass spectrometer (Shimadzu Co., Kyoto, Japan) equipped with a Nexera HPLC system (Shimadzu Co., Kyoto, Japan). For quantitative analysis, separation was carried out using Shim-pack XR-ODS II 3.0 mm i.d. × 75 mm, 2.2 µm maintained at 40°C. The mobile phase consisted of 10 mM ammonium acetate and acetonitrile at a flow rate of 0.4 mL/min. The gradient program for mobile phase B was as follows: 0 min, 3%; 0.25 min, 3%; 5 min, 95%; 7 min, 95%; 7.1 min, 3%; 10 min, 3%.

### Statistical analyses

Statistical analysis of IC concentrations was performed using GraphPad Prism (version 9.2.0, GraphPad Software, Inc., San Diego, California, United States). Paired *t* test was applied for comparison of time course changes in IC concentration. Differences in IC concentration at the same time point between control and UC subjects were assessed using unpaired *t* test or Mann‑Whitney *U* ‑test. Relationships between IC concentration and both clinical and endoscopic data were also assessed using Mann‑Whitney *U* ‑test. All values were expressed as the mean ± standard error of the mean or median and interquartile range. *P* < 0.05 was considered to indicate a statistically significant difference.

## Results

### Time course changes in serum IC level after spraying in the cecum

**Fig. 1:**
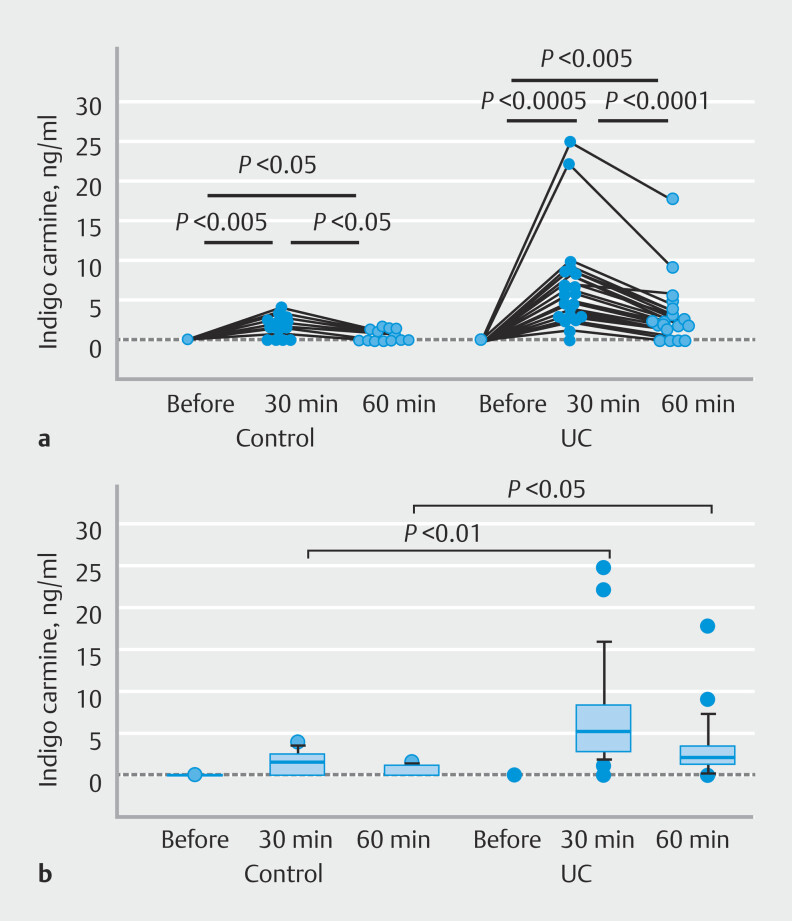
Time course changes in serum level of indigo carmine after spraying indigo carmine in the cecum. **a** All paired data are plotted. **b** Data are shown as median and interquartile range (IQR). Box plots were generated with the central box representing the IQR, containing the middle 50% of the data points. Lower and upper ends of the box correspond to the first quartile (Q1) and third quartile (Q3), respectively. The line within the box indicates the median of the dataset, dividing the data into two equal halves.

Characteristic of the patients enrolled are shown in Table 1 . To obtain basic information regarding changes in the serum IC level over time, we collected blood samples before spraying, and at 30 and 60 minutes after spraying. All patients in both the control and UC groups exhibited the highest level of IC at 30 minutes after spraying ( Fig. 1 **a** ). In both the control and UC groups, statistical analysis clearly showed that the IC level had a significant peak at 30 minutes and then decreased toward 60 minutes after spraying.

**Table:** **Table 1** Characteristics of control subjects and UC patients.

	**Control**	**UC**	***P* value **
Age (years)	62.0 ± 2.8 (49–79)	43.0 ± 3.2 (20–65)	0.0003
Sex (male/female)	7/5	16/4	NS
UC extension			
Total/left/rectal		17/2/1	
Clinical activity			
Remission/mild/moderate/severe		2/4/8/6	
MES at the cecum			
0/1/2		8/10/2	
Results as mean ± SE (range). Control, subject in screening of colon polyps. Clinical activity evaluated according to Mayo score. MES, Mayo endoscopic score; NS, not statistically significant; UC, ulcerative colitis.			

We next compared IC levels between controls and UC patients at the same time points. As shown in Fig. 1 **b** , the level was significantly higher in UC patients than in controls at both 30 and 60 minutes after spraying, suggesting that a larger amount of IC had permeated into the intestinal mucosa and moved into the vessels in UC patients.

### Relationship between serum IC level and endoscopic severity of cecal mucosa injury in patients with UC

**Fig. 2:**
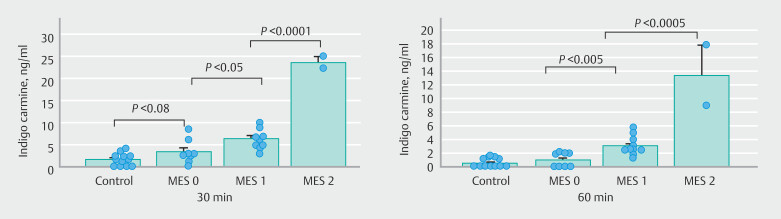
Relationship between serum indigo carmine level and endoscopic severity of mucosal injury in the cecum of patients with UC. MES, Mayo endoscopic subscore.

We also investigated the relationship between serum IC level and severity of cecal mucosa injury as evaluated by endoscopy ( Fig. 2 ). The IC level was significantly higher in MES 1 than in MES 0 patients at both 30 and 60 minutes after spraying. Similarly, it was significantly higher in MES 2 than in MES 1 patients at both 30 and 60 minutes after spraying. These data suggested that in UC patients, the permeability of IC was significantly promoted in mucosa with more severe injury.

### Relationship between serum IC level and clinical activity in patients with UC

**Fig. 3:**
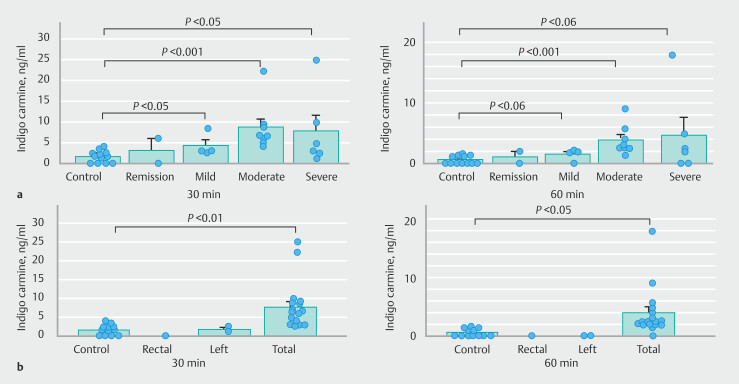
Relationship between serum indigo carmine level and **a** clinical activity or **b** extent of colitis in patients with UC. Clinical activity of UC was graded in terms of the Mayo score.

Although the serum IC level at 30 minutes tended to be higher in patients with moderate disease activity than in those with mild activity ( *P* = 0.198), the level did not differ significantly among patients with remission, mild, moderate, or severe activity at 30 or 60 minutes after spraying ( Fig. 3 **a** ). With regard to extension of disease, the IC levels at both 30 and 60 minutes were significantly higher in total-type than control but not different between left-side-type and total-type disease ( Fig. 3 **b** ). Blood biochemistry data are presented in **Supplementary Table 1** . The IC level at 30 minutes showed a positive correlation with white blood cell ( *P* = 0.022) and platelet counts ( *P* = 0.017), but no significant correlations were evident for other parameters.

## Discussion

Although leaky gut is considered to be a pivotal first step for development of harmful pathophysiology in many diseases 4 5 6 , few methods for evaluation of intestinal mucosal permeability in the living human body are available. In the present study, using peripheral blood samples, we could detect IC that had permeated into vessels from the intestinal lumen, suggesting that this new approach shows promise for evaluating permeability of the intestinal mucosa.

Although we could detect the permeated IC in blood, it is debatable whether the serum IC level actually reflects the degree of intestinal mucosal permeability. Accordingly, we selected patients with UC as positive controls, because it was expected that their intestinal permeability would be increased. As shown in Fig. 1, the serum IC level was clearly higher in UC patients than in controls at each of the measured time points, as we had expected. Moreover, in the UC group, we clearly showed that the IC level was significantly higher in patients with higher endoscopically assessed disease activity in the area where the dye had been sprayed. This finding suggested that the IC level was indeed correlated with severity of mucosal injury in the affected area.

It was also interesting that the IC level at 30 minutes tended to be higher in UC patients with MES0 (3.23 ± 0.94 ng/mL) than in the control group (1.58 ± 0.40 ng/mL) ( *P* = 0.08), although the difference was not significant due to the small number of subjects. This finding may suggest that our method would be useful for evaluation of intestinal permeability in patients with endoscopically undetectable mucosal injury, although this should be reconfirmed in a larger study. In addition, it is tempting to speculate that the peak IC level might occur earlier than 30 minutes in UC patients if their intestinal permeability is markedly high. In these contexts, we are planning to establish the best protocol for evaluation of intestinal permeability and reconfirm whether our method is able to detect increased intestinal mucosal permeability in areas that appear endoscopically normal in conditions such as irritable bowel syndrome or remission of UC.

Next, we also performed a subanalysis of the relationship between serum IC and clinico-endoscopic features. In relation to extent of colitis, the IC level was significantly higher in patients with total colitis than in those with left-sided colitis. This would seem to be reasonable because IC was sprayed in the cecum. Thus, inflammation and the accompanying increase in mucosal permeability in the cecum would be stronger in patients with total colitis. On the other hand, the IC level did not correlate with UC disease activity in terms of the Mayo score. This would also seem to be reasonable because the disease activity score is based on clinical features (stool frequency, rectal bleeding and physician rating of disease activity) and the grade of mucosal injury in the most severely damaged region of the colorectum. These findings strongly suggest that our method would be suitable for evaluation of intestinal permeability in a target area of the colonic mucosa. Because we sprayed IC only in the cecum, and not in other areas, in order to obtain precise data uninfluenced by unnecessary factors (differences in assessed area, disturbance of endoscopic observation/therapy, intestinal motility, etc.), we are planning to test our method in other areas of the gut and/or areas showing various degrees of mucosal injury.

To date, lactulose-mannitol test has been applied as a relatively acceptable assay for evaluation of intestinal mucosal permeability 15 16 . In this test, the patients orally receive lactulose and mannitol and collect their urine for 24 hours. Lactulose and mannitol are detected in urine collected for 24 hours after absorption from the intestinal lumen and excretion via the kidney. Therefore, the lactulose-mannitol test forces patients to restrict their movement, making it very inconvenient. Moreover, because lactulose/mannitol has to be administered orally, various factors including intestinal motility, contents, surgery or unidentified diseases in the gastrointestinal tract may affect efficacy of lactulose/mannitol absorption from the gastrointestinal lumen, thus decreasing its reliability as a test for intestinal mucosal permeability. In this context, our method allows evaluation of mucosal permeability in a target area, thus avoiding the above disturbance. In addition, our new method is very easy and convenient to perform during routine endoscopic examination, because IC is confirmed to be safe and no additional intervention is required. On the other hand, we have to discuss the comparison between our method and ex vivo testing of biopsies, which is another possible method to evaluate mucosal permeability. Ex vivo testing of biopsies requires special equipment such as a Ussing chamber, and biopsy samples are outside the human body, which is not a normal physiological condition. In this context, it may be debatable whether the obtained data reflect a real barrier function of the intestinal mucosa. Thus, we think that ex vivo testing of biopsies may have some weakness in evaluating physiological function of intestinal mucosal barrier in vivo compared with lactulose-mannitol test or our new method.

In addition to lactulose-mannitol test and ex vivo testing of biopsies, confocal laser endomicroscopy (CLE) recently has been highlighted as a useful tool to evaluating intestinal permeability. During CLE for evaluating intestinal permeability, the patients receive IV fluorescein and leakage of it is observed with a specific CLE probe positioned at the target point of intestinal mucosa. Thereafter, permeability is evaluated by estimating fluorescein leakage from the mucosal surface using specific software. The best advantage of CLE in evaluating mucosal permeability may be that it allows for in vivo assessment of mucosal permeability. Indeed, several studies using CLE have demonstrated that mucosal barrier dysfunction is associated with high risk of relapse of IBD and severity of IBS symptoms 17 18 19 20 . Furthermore Rath et al. have recently reported an interesting study in which mucosal permeability evaluated by CLE was more useful for predicting occurrence of major adverse outcomes compared with usual endoscopic findings 19 . In these contexts, we consider that evaluation of intestinal permeability is certainly important to advance the therapeutic strategy in the clinical setting of IBD and/or IBS. In regard to disadvantages, CLE and its related equipment is very expensive and not covered by medical insurance in our country, and CLE requires significant endoscopic skill because endoscopists have to determine the target position and precisely fix the CLE probe there during highly magnified observation. In addition, the evaluated permeability is focal but not regional in the intestinal lumen. In these contexts, our method is very easy in vivo assessment of mucosal permeability, and the evaluated permeability is considered to reflect regional but not focal permeability in the intestinal lumen.

## Conclusions

In summary, we have created a new method for evaluation of mucosal permeability with IC, a dye usually used during gastrointestinal endoscopy. This method has potential utility for assessment of mucosal permeability in target areas of the intestinal tract. On the other hand, we understand that this study has some limitations, such as sample size and heterogeneity of subjects in terms of age and type/severity of colitis. Patients in the UC group than the controls. This is due to the fact that UC patients are frequently young, whereas controls enrolled were older because they received colonic endoscopy for screening colon polyps. In addition, sample size might be a concern for clarifying the higher mucosal permeability in UC patients relative to the controls. In this regard, we will have to redesign this study in an age/sex/colitis-matched larger scale in the future. However, our main purpose in this study was not to clarify the higher mucosal permeability in UC but to establish a new method to evaluate intestinal mucosal permeability. In this context, we believe that our established method using endoscopically sprayed IC was at least successful for evaluating intestinal mucosal permeability. At present, although a few methods including lactulose-mannitol test, ex vivo testing of biopsies, and CLE are used for evaluating intestinal mucosal permeability, those methods have advantages and disadvantages, respectively. The advantage of our method may be that it makes in vivo assessment of mucosal permeability easier during routine endoscopic examination. However, we have to overcome some difficulties to make our method a popular standard for evaluating intestinal mucosal permeability. For example, the LC/MS assay for serum IC level is not available at any institutions at present. In addition, we have to improve the protocol for measuring serum IC level to be easier and more precise in a clinical setting. If these difficulties are resolved, our endoscopic methodology may have considerable potential for clarifying the underlying mechanism of “leaky gut” and/or contributing to assessment of clinical outcomes by evaluating intestinal mucosal permeability.

## Supplementary Material

Supplementary Material Zusatzmaterial
