# Supplementary material for: Endoscopic indigo carmine spraying for evaluation of intestinal mucosal permeability: Prospective pilot study
Source: Endosc Int Open. 2025 Sep 29;13:a26977599. doi: 10.1055/a-2697-7599 (PMC12509262; doi:10.1055/a-2697-7599)
Supplement: Supplementary file 1 — Supplementary Material [file 10-1055-a-2697-7599_26985853.pdf]

Supplementary Table 1 Blood biochemistry data for controls and UC patients.

Blood biochemistry	Control	UC	*P* value
WBC	5892 ± 615	8292 ± 923	0.082
Hb	13.9 ± 0.5	11.3 ± 0.6	0.005
Platelet	22.9 ± 1.4	36.4 ± 2.9	0.003
Albumin	4.2 ± 0.1	3.2 ± 0.2	< 0.0001
CRP	0.13 ± 0.05	1.68 ± 1.01	0.348
BUN	15.1 ± 0.9	12.9 ± 1.1	0.201
Creatinine	0.78 ± 0.04	0.82 ± 0.07	0.728
eGFR	69.1 ± 4.4	88.0 ± 6.0	0.040
AST	38.5 ± 16.8	21.8 ± 3.4	0.212
ALT	42.3 ± 22.8	47.7 ± 12.1	0.820

Data are presented as mean ± standard error.

ALT, alanine aminotransferase; AST, aspartate transaminase; BUN, blood urea nitrogen; CRP, C-reactive protein; eGFR, estimated glomerular filtration rate; Hb, hemoglobin; UC, ulcerative colitis; WBC, white blood cell.

Endosc Int Open | DOI 10.1055/a-2697-7599 | © The Author(s).
